# Delays in seeking abortion and its determinants among reproductive-age women based on the Ethiopian Demographic and health survey

**DOI:** 10.1016/j.heliyon.2024.e38477

**Published:** 2024-09-26

**Authors:** Asaye Alamneh Gebeyehu, Anteneh Mengist Dessie, Denekew Tenaw Anely, Melkamu Aderajew Zemene, Yilkal Negesse, Wondimnew Desalegn, Atitegeb Abera Kidie, Birtukan Gizachew Ayal, Angwach Abrham Asnake, Mulu Tiruneh, Assefa Agegnehu Teshome, Abebe Nega Zelelew, Getu Dessie Biru, Dejen Gedamu Damtie, Chalachew Yenew

**Affiliations:** a Department of Public Health, College of Health Sciences, Debre Tabor University, Debre Tabor, Ethiopia; b Department Public Health, College of Medicine and Health Sciences, Debre Markos University, Debre Markos, Ethiopia; c School of Public Health, College of Health Sciences, Woldia University, Woldia, Ethiopia; d Department of Epidemiology and Biostatistics, School of Public Health, College of Medicine and Health Sciences, Wolaita Sodo University, Wolaita Sodo, Ethiopia; e Department of Biomedical Science, College of Health Sciences, Debre Tabor University, Debre Tabor, Ethiopia; f Department of Statistics, CNS, Jimma University, Jimma, Ethiopia; g Department of Statistics, CNS, Dambi Dollo University, Dambi Dollo, Ethiopia

**Keywords:** Delayed seeking abortion, Abortion services, Pregnant women, Ethiopia

## Abstract

**Introduction:**

Delaying in seeking abortion services causes adverse public health outcomes, which increases maternal mortality rates due to complications from abortion. However, evidence shows that there are limited factors associated with delayed abortion. Therefore, the study aims to determine the magnitude and identify factors associated with delayed seeking abortion among reproductive-aged women in Ethiopia.

**Methods:**

Secondary cross-sectional data analysis was conducted on the Ethiopian Demographical and Health Survey dataset. A total weighted sample of 2198 reproductive-aged women was considered in this study. All data processing and statistical analyses were performed using R-software version 4.2.3. Multilevel binary logistic regression analysis was used to identify a significant predictor for delays in seeking abortion. An adjusted odds ratio with a 95 % confidence interval was used to measure a statistical association.

**Result:**

The prevalence of delays in seeking abortion among women in Ethiopia was 32 %. Educational status (AOR = 0.63; 95 % CI: 0.37–0.78), marital status (AOR = 2.12; 95 % CI: 1.38–3.74), wealth index (AOR = 0.54; 95 % CI: 0.41–0.75), frequency of listening a radio (AOR = 0.61; 95 % CI: 0.45–0.75), live births (AOR = 0.43; 95 % CI: 0.25–0.72), pregnancy wanted status (AOR = 0.59; 95 % CI: 0.4–0.79), contraceptive use (AOR = 0.46; 95 % CI: 0.34–0.69), and place of residence (AOR = 2.31; 95 % CI: 1.67–3.19) were statistically significantly variables associated with delays in seeking abortion.

**Conclusions:**

Delayed seeking abortion remains high in Ethiopia. The study is vital for policymakers, healthcare providers, and advocates to improve training on the importance of early abortion care, develop targeted interventions for better accessibility to abortion services, expand health education programs to raise awareness about reproductive rights and empower women with information for informed reproductive health decisions. Health education programs, specifically targeting uneducated women, poorer women, rural resident women, and women who do not use contraceptives, would decrease delays in accessing abortion services.

## 1 Introduction

Ethiopia has liberalized its abortion law under the Federal Democratic Republic of Ethiopia (FDRE) penal code to provide women with reproductive rights and choices of pregnancy termination services to have early, safe, and legal abortions [1]. Legalizing the abortion process could reduce substantial abortion-related maternal morbidity and mortality [2]. Despite the existence of abortion legislation, women encounter barriers when trying to access abortion care services, including discrimination and stigmatization in the community, opposition from healthcare providers, limited knowledge about abortion services, and a lack of trained providers and facilities, particularly in rural areas [[3], [4], [5]].

Abortion is time-restricted healthcare services that terminate the pregnancy before fetal viability, with suitable and early abortion performed up to and including 12 weeks of gestation [6]. Abortion is a common practice and can be performed up to 28 weeks of gestation in Ethiopia, such as in the case of rape, incest, socio-economic difficulty, unwanted pregnancy conditions, and the reason for health-related risks to pregnant women or fetuses [3,7]. Abortion after 20 weeks of gestation may be permitted only in exceptional circumstances. The early timing of abortion is a critical element in determining clinical outcomes, including shorter abortion intervals, lower incomplete abortion rates, and reduced side effects to optimize patient outcomes and minimize complications [8].

Abortion cases have increased substantially due to increased availability and accessibility to abortion-care services [9]. Globally, an estimated 55.9 million abortions occur each year, with 49.3 million abortions in developing countries and 6.6 million in developed countries [10]. An estimated 620,300 abortion cases occurred in Ethiopia, with an annual abortion rate of 28 per 1000 among women aged 15–49 [9]. Second-trimester abortion (after 12 weeks of gestation) accounts for over 23 % of abortions performed in Ethiopia [11,12], 10.3 % in the United States [13], 13.6 % in Mexico [14], 15.3 % in Zambia [15], 25 % in South Africa [16], and 46 % in Kenya [17]. Abortion in the second trimester is associated with higher risks of complications and deaths compared to first-trimester abortion [11].

Abortion care services are essential to improve reproductive health; however, many pregnant women may delay accessing and seeking abortion health services [8,18,19]. Delays in seeking abortion are a major public health problem that affects future maternal health and health outcomes [19]. Delays in seeking abortion (abortion occurs after 12 weeks of gestation) have significantly contributed to higher maternal morbidity and mortality, increased costs to healthcare services, increased risks of abortion-related complications, and also decreased feasibility of abortions [20]. Delayed abortion can indeed limit reproductive healthcare service options and can negatively affect women's emotional, physical, psychological, and social well-being both in the present and future life [5,21].

Previous studies have identified several factors associated with delay in seeking abortion, such as socio-demographic, socio-economical, and obstetric-related characteristics [17,19,[22], [23], [24], [25]]. To our knowledge, no previous studies on delayed seeking of abortion have utilized nationally representative data, nor have they conducted multilevel analyses to identify associated factors Therefore, this study aimed to identify factors associated with delays in seeking abortion among reproductive-aged women based on the 2016 Ethiopian Demographic and Health Survey using multilevel analysis.

## 2 Methods

### 2.1 Study design and data sources

We conducted the study based on the cross-sectional secondary data of the EDHS 2016 dataset, which was collected from October 2017 to March 2018 [26]. The EDHS-2016 survey used two-stage stratified random sampling to select the representative samples and to collect the data. The survey was conducted in Ethiopia's geographical regions (Tigray, Afar, Amhara, Oromia, Somali, Benishangul, SNNPR (south nation nationalities people's region), Gambella, and Harari) and two administrative cities (Addis Ababa and Dire Dawa). Each region was divided into urban and rural areas, and the enumeration area (EAs) samples were independently selected from each stratum. The EDHS-2026 survey used a women's questionnaire to collect demographic characteristics and sexual and reproductive health indicators (marriage, pregnancy, fertility, family planning, sexual behavior, maternal health, STIs, and HIV/AIDS). For this study, data were extracted from the Birth Record file (BR) of the standard DHS dataset. In this study, the source populations consisted of all reproductive-aged women who had undergone abortion in the five years preceding the survey, while the study population comprised all reproductive-aged women who experienced abortion in the selected cluster. After removing any missing data related to the time of abortion, a final weighted sample size of 2198 reproductive-aged women who had undergone abortions was included in the analysis.

### 2.2 Study variables

#### 2.2.1 Outcome variable

The outcome variable for this study was delayed abortion seeking among women of reproductive age. The outcome variable was classified as “yes” if the abortions occurred after 12 weeks of pregnancy, Otherwise “no” if the abortions occurred before or at 12 weeks [27].

#### 2.2.2 Explanatory variables

The explanatory variables for this study contain individual-level and community-level variables. Individual-level variables include sociodemographic and obstetric-related characteristics. Sociodemographic characteristics (age, wealth index, women's educational level, partner's educational level, marital status, and women's occupational status), obstetric-related characteristics (live-birth history, pregnancy wanted status, contraceptive use), and community-level variables (residence and region) were explanatory variables. The demographic and health survey program computed the household wealth index using principal component analysis, and the wealth index is categorized into five quantiles such as poorest, poor, middle, rich, and richest. For the current study, we grouped household wealth indices into three quantiles: poor (combining poor and poorest), middle, and rich (combining rich and richest). Based on EDHS recoding variables, we considered four categories of educational status of women or their partners (No education, primary, secondary, and higher education). We categorized contraceptive use measurement into two groups based on the utilization status of any contraceptive methods to delay or avoid pregnancy, such as women who use any contraceptive methods and those who do not use any contraceptive methods. The measurement of the frequency of listening to the radio, watching television, or reading newspapers indicates frequent exposure to any family planning programs (Not at all, less than once a week, and at least once a week) [28].

### 2.3 Data management and statistical analysis

All statistical analyses were conducted using R 4.2.3 software. Before conducting all statistical analysis, data were weighted employing sampling weights, sampling primary units, and strata to get valid statistical parameter estimates by restoring the national representativeness of the survey. Descriptive statistics (frequency and percentage) describe the study population characteristics. A multilevel approach depicts that the delays to abortion variations observed in the binary outcome are nested in clusters. Suppose that $Y_{ij}$ represents $i^{\text{th}}$ women experiencing delays in seeking abortion in $j^{\text{th}}$ clusters and also $P_{\text{ij}}$ represents the probability of delays in seeking an abortion for $i^{\text{th}}$ women in $j^{\text{th}}$ clusters. The mathematical equation of multilevel binary logistic regression analysis can be described using logit link function as below:

$$\text{Logit}\left[\frac{P_{ij}}{1-P_{ij}}\right]=\beta_{0}+\beta X_{ij}+U_{j}$$

where $\beta_{0}$ denotes the overall intercept, β the vector of regression coefficients, $X_{ij}$ the vectors of independent variables, and $U_{j}$ the random effect at cluster-level [29].

The predicted probability of delayed seeking abortion among women can be calculated below as:

$$P_{\text{ij}}=\frac{\exp\left({X_{ij}}^{\prime }\beta +U_{j}\right)}{1+\exp\left({X_{ij}}^{\prime }\beta +U_{j}\right)}$$

We conducted a multilevel binary logistic regression analysis to identify factors associated with delays in seeking abortion. First, we performed bivariable analysis to identify potential candidate factors for delayed abortion seeking, considering variables with a p-value less than 0.25 for multivariable analysis [30]. In the multivariable analysis, we reported adjusted odds ratios with 95 % confidence intervals to measure the observed association, and these variables with p-values less than 0.05 were considered statistically significant factors associated with delays in seeking abortion.

We have fitted four different models by considering individual and community-level variables: the first null model with no explanatory variables, the second model contains only individual-level variables, the third model with only community-level variables, and finally, the fourth model includes both individual-level and community-level variables in the analysis. We used deviance criteria to choose the better-fitted model, and the model with the lowest deviance value from these four models was selected as a better-fitted model with data. The variance variability in delays to abortion was measured using the intra-correlation coefficient (ICC), median odds ratio (MOR), and proportional change in variance (PCV). The ICC describes the cluster variability, which indicates the total proportional variance in the delays to abortion is attributable to the cluster level. The MOR is the median value of odds ratios between an individual at a higher risk cluster and a lower risk cluster of delays to abortion when randomly picked out of two clusters, and also shows unexplained heterogeneity between clusters. The PCV measures the total explained variation in delayed abortion seeking attributable to including individual and community-level variables in the model.

### 2.4 Ethical consideration

This study used secondary data, so it does not require further ethical approval. Data were accessed and downloaded from the publicly available domestic DHS program website (https://www.dhsprogram.com) by submitting the aim of the study through online registration. All methods for this study were performed following the declaration of Helsinki.

## 3 Result

### 3.1 Prevalence of delayed seeking abortion by respondents’ characteristics

The study included a total weighted sample size of 2198 reproductive-aged women. Among 2198 reproductive-aged women, almost 1989 (90.5 %) women were residents of rural areas, 33.2 % of those women experienced delays in seeking an abortion, while 209 (9.5 %) were residents of urban areas, 20.1 % of those were delayed abortion seeking. About 557 (25.3 %) of the respondents were women aged 35–39 years, and 45.9 % of the highest prevalence of delayed seeking abortion was observed for women aged 45–49 (Table 1).

**Table 1:** Prevalence of delayed seeking abortion and respondents’ characteristics in Ethiopia, EDHS 2016 (N=2198).

Variables	Women	Abortion seeking status	
		Delay	Early
	n (%)	n (%)	n (%)
**Age (years)**			
15–19	40 (1.8)	18 (45.0)	22 (55.0)
20–24	139 (6.3)	46 (33.1)	93 (66.9)
25–29	361 (16.4)	130 (36.0)	231 (64.0)
30–34	511 (23.2)	138 (27.0)	373 (73.0)
35–39	557 (25.3)	145 (26.0)	412 (74.0)
40–44	409 (18.6)	143 (35.0)	266 (65.0)
45–49	181 (8.2)	83 (45.9)	98 (54.1)
**Marital status**			
Unmarried	81 (3.7)	22 (27.5)	59 (72.5)
Married	2117 (96.3)	681 (32.2)	1436 (67.8)
**Religion**			
Orthodox	761 (34.6)	324 (42.6)	437 (57.4)
Muslim	500 (22.7)	105 (21.1)	395 (78.9)
Protestant	937 (42.6)	274 (29.2)	663 (70.8)
**Educational levels**			
No education	1452 (66.1)	475 (32.7)	977 (67.3)
Primary	603 (27.4)	183 (30.3)	420 (69.7)
Secondary	81 (3.7)	26 (32.1)	55 (67.9)
Higher	62 (2.8)	19 (30.6)	43 (69.4)
**Partner educational levels**			
No education	952 (43.3)	342 (35.9)	610(64.1)
Primary	1004 (45.7)	281 (28.0)	723 (72.0)
Secondary	146 (6.6)	60 (41.2)	86 (59.8)
Higher	96 (4.4)	20 (20.8)	76 (79.2)
**Place of residence**			
Urban	209 (9.5)	42 (20.1)	167 (79.9)
Rural	1989 (90.5)	661 (33.2)	1328 (66.8)
**Region**			
Tigray	159 (7.2)	64 (40.3)	95 (59.7)
Afar	85 (3.9)	22 (25.9)	63 (74.1)
Amhara	327 (14.9)	181 (55.4)	146 (44.6)
Oromia	612 (27.8)	179 (29.2)	433 (70.8)
Somali	86 (3.9)	21 (24.4)	65 (75.6)
Benishangul-Gumuz	88 (4.0)	24 (27.3)	64 (72.7)
SNNPs	340 (15.5)	86 (25.3)	254 (74.7)
Gambella	108 (4.9)	27 (25.0)	81 (75.0)
Harari	130 (5.9)	35 (26.9)	95 (73.1)
Dire Dawa	122 (5.6)	25 (20.5)	97 (79.5)
Addis Ababa	141 (6.4)	39 (27.7)	102 (72.3)
**Wealth index**			
Poor	974 (44.3)	378 (38.8)	596 (61.2)
Middle	825 (37.5)	207 (25.2)	618 (74.8)
Rich	399 (18.1)	118 (29.6)	281 (70.4)
**Frequency of listening to a radio**			
Not at all	1528 (69.5)	558 (36.5)	970 (63.5)
Less than once a week	209 (9.5)	56 (26.9)	153 (26.9)
At least once a week	461 (21.0)	89 (19.3)	372 (80.7)
**Frequency of watching television**			
Not at all	1749 (79.5)	584 (33.4)	1163 (66.4)
Less than once a week	290 (13.2)	58 (20.1)	232 (79.9)
At least once a week	161 (7.3)	61 (38.1)	100 (61.9)
**Occupational status**			
Unemployed	1095 (49.8)	254 (23.2)	841 (76.8)
Employed	1103 (50.2)	449 (40.7)	654 (59.3)
**Live birth history**			
No births	595 (27.1)	261 (43.9)	334 (56.1)
One births	915 (41.7)	247 (27.1)	668 (72.9)
Two births	467 (21.2)	143 (30.6)	324 (69.4)
Three and more births	221 (10.0)	72 (32.6)	149 (67.4)
**Preceding birth intervals**			
Less than 24 months	587 (26.7)	161 (27.4)	426 (72.6)
≥24 months	1611 (73.3)	542 (33.6)	1069 (66.4)
**Pregnancy desired**			
Wanted then	144 (6.5)	53 (36.8)	91 (63.2)
Wanted Later	750 (34.1)	296 (38.9)	464 (61.1)
Never wanted	1304 (59.4)	354 (27.1)	950 (72.9)
**Smoking status**			
Yes	43 (1.9)	13 (30.2)	30 (69.8)
No	2155 (98.1)	690 (32.0)	1465 (68.0)
**Contraceptive use**			
Yes	482 (21.9)	186 (38.6)	296 (61.4)
No	1716 (78.1)	517 (30.1)	1199 (69.9)
Total	2198 (100)	703 (32.0)	1495 (68.0)

The majority of the respondents, 2117 (96.3 %) of women were married, and the prevalence of delayed abortion among those women was 27.5 %. Nearly two-thirds, 1452(66.3 %) of the respondents had no education, 603 (27.4 %) had primary education, and 62 (2.8 %) had higher education. The highest prevalence of delayed seeking an abortion was observed for women with no education (32.7 %) (Table 1).

Amongst 947 women from a poor wealth index, 38.8 % experienced a higher prevalence of delayed seeking abortion. The majority of women, 1528 (69.5 %), reported never listening to the radio, while 1749 (79.5 %) never watched television. The proportion of delays in seeking an abortion was highest among women who never listened to the radio (36.5 %) and women who watched the television at least once a week (38.1 %). Thirty-eight percent of the women who use contraceptive methods experienced delayed seeking abortion (Table 1).

### 3.2 Magnitude of delays in seeking abortion among women in Ethiopia, EDHS 2016

In this study, the prevalence of delayed seeking abortion among reproductive-aged women was 32 % (703). The 95 % confidence interval of the prevalence of delays in seeking abortion was statistically found between 30 % and 34 % (95 % CI: 30–34) (Table 1).

### 3.3 Factor associated with delays in seeking abortion among reproductive-aged women

#### 3.3.1 Random effects and model selection

The null model revealed that 39.2 % of the total variation in delayed seeking abortion is attributable to cluster-level or community-level factors. The median odds ratio in the null model was 3.98, indicating that women in a cluster with a higher risk of delays in seeking abortion had 3.98 times higher odds of experiencing delayed seeking abortion compared to women in a cluster area with a lower risk of delays in seeking abortion. The highest PCV value (42 %) of delayed seeking abortion in the last model accounted for 42 % of the total variance explained by the inclusion of both individual and community-level factors. The final model (Model IV) was chosen as the best-fitted model because it has the lowest deviance value of 1239.9 (Table 2).

**Table 2:** Cluster variability and multivariable multilevel logistic regression for identifying factors of delays in seeking abortion among reproductive-aged women in Ethiopia, 2016.

Variables	Model1	Model II	Model III	Model IV
**Age**				
15–19		Ref		Ref
20–24		0.95(0.32–2.83)		0.80(0.28–2.26)
25–29		1.46(0.49–4.28)		1.19(0.43–3.34)
30–34		1.48(0.50–4.42)		1.15(0.40–3.26)
35–39		1.78(0.59–5.41)		1.28(0.44–3.65)
40–44		1.77(0.61–5.14)		1.66(0.54–5.14)
45–49		1.49(0.49–4.51)		1.42(0.44–4.62)
**Educational status**				
No education		Ref		Ref
Primary		0.91(0.69–1.20)		0.84(0.46–1.54)
Secondary		0.53(0.30–0.94)^a^		0.44(0.29–0.9)^a^
Higher		0.74(0.57–0.89)^a^		0.63(0.37–0.78)^a^
**Wealth index**				
Poor		Ref		Ref
Middle		0.82(0.61–1.10)		0.88(0.64–1.22)
Rich		0.65(0.49–0.87)^a^		0.54(0.41–0.75)^a^
**Marital status**				
Unmarried		Ref		Ref
Married		2.26(1.31–3.90)^a^		2.12(1.38–3.74)^a^
**Maternal occupation**				
Unemployed		Ref		Ref
Employed		1.24(1.02–1.51)^a^		1.09(0.87–1.36)
**Frequency of listening to a radio**				
Not at all		Ref		Ref
Less than once a week		1.36(0.96–1.94)		1.14(0.91–1.86)
At least once a week		0.67(0.39–0.87)^a^		0.61(0.45–0.75)^a^
**Frequency of watching television**				
Not at all		Ref		Ref
Less than once a week		0.82(0.54–1.24)		0.55(0.36–1.26)
At least once a week		0.56(0.37–0.83)^a^		0.36(0.27–0.67)^a^
**Pregnancy wanted status**				
Wanted then		Ref		Ref
Wanted later		0.62(0.29–1.06)		0.66(0.32–1.32)
Never wanted		0.73(0.47–0.94)^a^		0.59(0.4–0.79)^a^
**Live birth history**				
No birth		Ref		Ref
One birth		0.81(0.60–1.10)		0.62(0.57–1.51)
Two births		0.73(0.57–0.92)^a^		0.65(0.46–0.9)^a^
Three and more births		0.59(0.36–0.85)^a^		0.43(0.25–0.72)^a^
**Contraceptive use**				
Yes		0.57(0.37-0.73)^a^		0.46(0.34–0.69)^a^
No		Ref		Ref
**Residence**				
Urban			Ref	Ref
Rural			3.44(2.21–5.36)^a^	2.31(1.67–3.19)^a^
**Region**				
Tigray			1.95(0.83–4.53)	3.31(0.33–5.21)
Afar			5.38(2.38–12.2)^a^	4.18(3.02–7.10)^a^
Amhara			7.82(3.41–17.9)^a^	6.31(3.90–7.22)^a^
Oromia			4.05(2.32–7.83)^a^	4.43(1.73–4.49)^a^
Somali			6.9(3.13–10.3)^a^	6.5(5.78–8.6)^a^
Benishangul-Gumuz			6.0(2.54–9.2)^a^	5.7(4.14–7.4)^a^
SNNPs			4.3(1.75–8.5)^a^	4.4(2.23–6.17)^a^
Gambella			5.9(3.76–9.1)^a^	5.3(2.54–7.62)^a^
Harari			3.54(0.67–7.65)	3.46(0.66–6.2)
Dire Dawa			4.62(0.93–6.42)	3.54(0.84–5.12)
Addis Ababa			Ref	Ref
Cluster variability				
Variance	2.12	1.38	1.65	1.23
ICC	39.2	29.5	33.4	27.2
PCV	Ref	34.9	22.2	42.0
MOR	3.98	3.05	3.38	2.86
Deviance	1499.7	1299.6	1447.9	1239.9

Ref: reference.

a p-value <0.05 with 95% confidence interval (significant).

In the multiple multilevel logistic analysis, educational status, wealth index, marital status, frequency of listening to the radio, frequency of watching television, live-birth history, contraceptive use, residence, and region were variables statistically significantly associated with delays in seeking abortion.

Women with secondary education were 56 % less likely to seek delayed abortion than those with no education, and women with higher education had 37 % (AOR = 0.63; 95CI: 0.37–0.78) lower odds of seeking delayed abortion than those with no education. Women living in rural areas were 2.31 times (AOR = 2.31; 95 % CI: 1.67–3.19) more likely to seek delayed abortion than those living in urban areas. The odds of experiencing delayed seeking abortion among women from a rich wealth index were 0.54 times lower than those from a poor wealth index. Married women were 2.12 (AOR = 2.12; 95 % CI: 1.38–3.74) times more likely to seek delayed abortion than those unmarried women. The likelihood of seeking delayed abortion among women who listened to a radio at least once a week was 39 % (AOR = 0.61; 95 % CI: 0.45–0.75) times lower than those women who never listened to a radio, whereas women who watched television at least once a week were 64 % less likely to seek delayed abortion than those never watched television. Women who had two live births and three or more live births were 35 % (AOR = 0.65: 95 % CI: 0.46–0.90) and 57 % (AOR = 0.43: 95 % CI: 0.25–0.72) less likely to experience delayed seeking abortion than women who had no live births. Women who never wanted their pregnancy status were 41 % (AOR = 0.59; 95 % CI: 0.4–0.79) less likely to seek delayed abortion than those who desired their pregnancy status then. The odds of experiencing delayed abortion seeking among women who used contraceptive methods were 54 % (AOR = 0.46: 95 % CI: 0.34–0.69) lower than those who never used contraceptive methods (Table 2).

## 4 Discussion

Delayed abortion is a major public health problem, causing maternal morbidity and mortality in Ethiopia [11]. The study aimed to determine the magnitude and identify the determinants of delays in seeking abortion among reproductive-aged women in Ethiopia. The study found that the prevalence of delay in seeking abortion was 32 %. Accordingly, educational status, wealth index, marital status, frequency of listening to the radio, frequency of watching television, pregnancy desired status, birth history, residence, and region were statistically significantly associated with delays in seeking an abortion.

In this study, the prevalence of delays in seeking abortion among reproductive-aged women in Ethiopia was 32 % (95 % CI: 30–34 %), which is higher than the study conducted in Ara Minch and Wolayita Sodo, South Ethiopia (23 %) [11] Amhara region, Ethiopia (19.2 %) [31], and Debre Markos, Ethiopia (29.6 %) [32]. The difference may be due to the study considering national figures, including more data from rural areas with limited healthcare access, resulting in higher delays in seeking abortion. Additionally, stronger cultural or religious opposition to seeking abortion, as well as the cost and availability of transport services to health facilities, can also serve as significant barriers to seeking abortion early [33,34]. However, the prevalence of delays in seeking abortion among women in Ethiopia was lower than the study conducted in Kenya (39 %) [35]. The possible reason for such observed discrepancy might be the combination of factors related to healthcare systems, policy and legal environments, socio-cultural norms, economic conditions, health educational interventions, and the overall effectiveness of the health system [36,37]; with improvements in maternal healthcare services availability and accessibility can also significantly impact health outcomes, potentially reducing the risks of delayed abortion over time. The observed difference implies that variability in the availability of abortion services, geographical disparities, differences in abortion laws and regulations, national policies, healthcare infrastructure, and cultural attitudes on the timeliness of seeking abortion services [38,39].

More educated women were less likely to seek delayed abortion than women with no education. This study is consistent with the studies done in Kenya, Nigeria, and Ethiopia [19,23,35]. The possible reason could be that educated women often have better access to information about reproductive health services, including contraception and abortion services. They may also have greater awareness of their reproductive rights and available healthcare options, making them more likely to seek abortion services promptly if they find themselves facing an unwanted pregnancy [40,41]. Furthermore, educated women tend to have more autonomy and decision-making power over their bodies and reproductive choices to navigate the healthcare system and advocate for their own needs, leading to more timely access to abortion services [42].

Our study showed that the wealth index had a negative association with delays in seeking abortion. Women from wealthy households had decreased the likelihood of delays in seeking abortion. This finding is consistent with the previous studies conducted in Ghana, United States, and India [22,[42], [43], [44]]. Wealthy women might have improved access to quality healthcare services, comprehensive reproductive health education, and better financial resources, which can help those women make timely decisions about their reproductive health and access timely medical care, contributing to lower delays in seeking abortion [45,46]. Moreover, wealthy women may have financial resources to cover the cost of accessing better maternal healthcare services. In contrast, women with a lower wealth index may face barriers to accessing safe abortion services due to costs such as transportation expenses [47]. Financial barriers are common issues for abortion-seeking women of lower socioeconomic status, who are experiencing more delays in each step of the abortion process. Place of residence was another significant independent variable that positively affected delays in seeking abortion. Women living in rural areas were more likely to delay in seeking abortion. This study finding is in line with the studies done in Nigeria and Ethiopia [23,31,48]. The reason for this is that women living in rural areas are more likely to delay seeking abortion due to a lack of adequate information about where to access appropriate services and the limited availability of sexual and reproductive health services [49]. This lack of access can result in significant delays in seeking abortion services, which can have serious adverse outcomes for women's health and well-being. In addition, cultural norms and religious restrictions in rural communities can make it difficult for women to seek abortion services, indicating women feel ashamed, afraid of being seen by others, and worried about the lack of privacy and confidentiality of health providers when seeking timely abortion services [50]. So, it is crucial to ensure that women in rural areas have the essential health information and services they need to make timely and informed decisions about their reproductive health and abortion services.

Married women had higher odds of experiencing delays in seeking abortion than unmarried women. This is in line with the studies conducted in Ethiopia [25]. This could be because married women may face several challenges in timely accessing abortion services, such as financial constraints, lower self-autonomy in decision-making, social pressures, and fears of judgment from partners or communities [51]. Married women who begin childbearing at an older stage do not use contraceptive methods to have multiple children they need, so women confirm their pregnancy late and are not well-familiar with family planning methods like accessing abortion services to terminate unwanted pregnancies [52]. This study revealed that the frequency of listening to the radio and the frequency of watching television was a statistically significant variable associated with delays in seeking an abortion. Women exposed to mass media (listening to radio and watching television) were less likely to delay in seeking abortion. The finding of this study is consistent with the other studies in Ghana and Mozambique [53]. Social media plays a significant role in creating awareness of the benefits of timely accessing sexual reproductive healthcare services to improve reproductive health. The possible reason could be mass media exposed women are often more aware of abortion care services, know where to get family planning methods, easily access comprehensive information about the available options, and reduce fear of abortion stigma [54]. Exposure to mass media can provide women with more knowledge and resources to make informed decisions about their reproductive health, leading to decreased delays in seeking abortion services.

In this study, pregnancy desired status was a statistically negative association with delays in seeking abortion. Women who never wanted to continue their pregnancy had lower odds of delays in seeking abortion. This study result is similar to the studies done in Ethiopia and Kenya [31,35,55]. Women who have no desire to continue their pregnancies at all may not face difficulty in making decisions about whether or not to terminate pregnancies. Women who do not want to continue their pregnancies may not be psychologically and emotionally well prepared to continue their pregnancy because they are facing economic or financial problems and unstable living conditions [56]. The possible reason might be those women who do not desire to continue the pregnancy at all confirm their pregnancy test earlier before the fetus grows and do not delay in deciding to terminate the pregnancy. The number of live births was also found to be another significant variable associated with delays in seeking abortion. Women who had two and three or more live births had lower odds of seeking delayed abortions than women who had no live births. The finding is consistent with the previous studies in Nepal, Kenya, and Ethiopia [25,35,40,57]. The possible reason could be women who have more children may have an enhanced understanding of their sexual and reproductive health to address unplanned pregnancies that may increase family sizes in the household. Moreover, women having previous experiences with childbirth and parenting may prompt them to make timely decisions regarding their reproductive choices, resulting in reduced delays in seeking abortion services [57].

The current study examined the relationship between contraceptive use and delayed abortion among reproductive-aged women. Women who used contraceptive methods were less likely to delay seeking abortion than women who never used contraceptive methods. This is consistent with the study conducted in Ethiopia, Kenya, and the United States [24,58,59]. The possible reason may be women using contraceptives are often doing so to prevent unintended pregnancies. This proactive approach suggests a commitment to avoiding unwanted pregnancies and may reduce the likelihood of delay in seeking abortion if contraception fails [60]. Contraceptive users are generally more aware of family planning methods, reproductive health, and overall reproductive rights to make informed decisions about their health and well-being and also contribute to the overall well-being of families and communities. Consequently, this awareness may also lead to early recognition of pregnancy, prompting timely decisions and actions and accessing early healthcare services, reducing delays in seeking abortion. Based on the geographical region, there was a vital significant variability in seeking delayed abortion across the regions in Ethiopia.

## 5 The strengths and limitations of the study

The strength of this study was derived from nationally representative survey data in Ethiopia, making generalizability to the entire population. However, the study has limitations, as the cross-sectional nature of the dataset makes it difficult to establish causal relationships. There might be recall bias because of the reproductive women's report. Some relevant variables, such as attitudes about abortion care, emotional factors, severity of complications, and pregnancy symptoms, were not included in the study because the data was not available in the survey collection.

## 6 Conclusion

The prevalence of delays in seeking abortion in Ethiopia remains high, with nearly one-third of women experiencing delayed abortion seeking. In the multilevel binary logistic regression model, educational status, pregnancy wanted status, wealth index, frequency of listening to the radio, frequency of watching television, live birth history, and contraceptive use are statistically negatively associated with delays in seeking abortion; on the other hand, marital status, place of residence, and region have statistically significant positive associations with delays in seeking abortion. The key findings can facilitate collaboration for evidence-based strategies, prioritize timely abortion care, and empower women with resources for informed reproductive health decisions. These can also foster a supportive environment that ensures equitable access to reproductive healthcare services and promotes women's autonomy in making choices related to their reproductive lives. Increasing health education and improving accessibility of family planning options in the communities should target uneducated women, poorer women, rural resident women, and women who do not currently use contraceptives that help them prevent themselves from unplanned pregnancies and provide early access to abortion services. Therefore, these results are vital for policymakers and designers to improve health interventions for women who have delayed early pregnancy termination and undergo unsafe abortions.

## Ethics approval and consent

Data set for this study were obtained from the DHS program. We accessed and downloaded the data set after describing the purpose of the study in the online platform. The data sets obtained from the DHS program are kept confidential, and excluded participant's personal identifiers. The research is conducted according to the declaration of Helsinki.

## Consent for publication

Not applicable.

## Data and material availability

The data set is accessed from publicly available website (https://www.dhsprogram.com). The data set used and/or analyzed during the current study is available from the corresponding author on reasonable request.

## Funding

There is no declared funding for conducting this study.

## CRediT authorship contribution statement

**Asaye Alamneh Gebeyehu:** Writing – review & editing, Writing – original draft, Visualization, Validation, Software, Methodology, Investigation, Formal analysis, Data curation, Conceptualization. **Anteneh Mengist Dessie:** Writing – review & editing, Writing – original draft, Visualization, Validation, Methodology, Investigation, Formal analysis, Data curation, Conceptualization. **Denekew Tenaw Anely:** Writing – review & editing, Writing – original draft, Visualization, Validation, Methodology, Investigation, Data curation, Conceptualization. **Melkamu Aderajew Zemene:** Writing – review & editing, Writing – original draft, Visualization, Validation, Methodology, Investigation, Data curation, Conceptualization. **Yilkal Negesse:** Writing – review & editing, Writing – original draft, Visualization, Validation, Methodology, Investigation, Data curation, Conceptualization. **Wondimnew Desalegn:** Writing – review & editing, Writing – original draft, Visualization, Validation, Methodology, Investigation, Data curation, Conceptualization. **Atitegeb Abera Kidie:** Writing – review & editing, Writing – original draft, Visualization, Validation, Methodology, Investigation, Data curation, Conceptualization. **Birtukan Gizachew Ayal:** Writing – review & editing, Writing – original draft, Visualization, Validation, Methodology, Investigation, Data curation, Conceptualization. **Angwach Abrham Asnake:** Writing – review & editing, Writing – original draft, Visualization, Validation, Methodology, Investigation, Data curation, Conceptualization. **Mulu Tiruneh:** Writing – review & editing, Writing – original draft, Visualization, Validation, Methodology, Investigation, Data curation, Conceptualization. **Assefa Agegnehu Teshome:** Writing – review & editing, Writing – original draft, Visualization, Validation, Methodology, Investigation, Data curation, Conceptualization. **Abebe Nega Zelelew:** Writing – review & editing, Writing – original draft, Visualization, Validation, Methodology, Investigation, Data curation, Conceptualization. **Getu Dessie Biru:** Writing – review & editing, Writing – original draft, Visualization, Validation, Methodology, Investigation, Data curation, Conceptualization. **Dejen Gedamu Damtie:** Writing – review & editing, Writing – original draft, Visualization, Validation, Methodology, Investigation, Data curation, Conceptualization. **Chalachew Yenew:** Writing – review & editing, Writing – original draft, Visualization, Validation, Methodology, Investigation, Data curation, Conceptualization.

## Declaration of competing interest

The authors declare that they have no known competing financial interests or personal relationships that could have appeared to influence the work reported in this paper.

## Abbreviations and acronyms

AOR: Adjusted Odds ratio
CI: Confidence Interval
EA: Enumeration Area
EDHS: Ethiopian Demographic and Health Survey
EHNR: Ethiopian Health Nutrition and Research Institute (EHNRI)
ICC: Intra-Correlation Coefficient
MOR: Median Odds Ratio
(NRERC): National Research Ethics Review Committee
PCV: proportional change in variance
STIs: Sexually Transmission Infections
WHO: World Health Organization
